# Functional similarity of ABP 959 and eculizumab in simulated serum models of aHUS and NMOSD

**DOI:** 10.1007/s00277-023-05439-4

**Published:** 2023-10-10

**Authors:** Helen J. McBride, Ashley Frazer-Abel, Sandra Thiemann, Sonya G. Lehto, Katariina M. Hutterer, Jennifer Liu

**Affiliations:** 1 Amgen Inc., One Amgen Center Dr., Thousand Oaks, CA 91320 USA; 2 School of Medicine, University of Colorado, Aurora, CO 80045 USA

**Keywords:** Biosimilar, Eculizumab, ABP 959, Atypical hemolytic uremic syndrome, Neuromyelitis optica spectrum disorder, Similarity

## Abstract

ABP 959 is being developed as a biosimilar to Soliris® (eculizumab) reference product (RP), which was approved under orphan designation for a group of rare diseases including paroxysmal nocturnal hemoglobinuria (PNH), atypical hemolytic uremic syndrome (aHUS), generalized myasthenia gravis (gMG), and neuromyelitis optica spectrum disorder (NMOSD). Development of biosimilars for therapeutics approved for rare disease indications must provide scientific rationale based on the totality of evidence (TOE). To support the TOE and the scientific justification for extrapolation to all approved indications for eculizumab RP, including but not limited to aHUS and NMOSD, we utilized simulated ex-vivo pharmacodynamic (PD) assessments to compare the complement component 5 (C5) inhibitory activity of ABP 959 and the RP. Hemolysis activity of CH50 and AH50, and Wieslab CP, AP, and LP endpoints represent the three complement activation pathways (classical, alternative, and lectin), all of which share the terminal pathway and require C5 for activity. These endpoints were evaluated in normal serum, simulated aHUS serum, and simulated NMOSD serum to provide a robust comparison. The results support the conclusion that ABP 959 and eculizumab RP exhibit highly similar inhibition of C5 function regardless of the type of serum used. This work presents a full comparison of the effect of C5 inhibition across five complement functional assays. Using this approach to confirm functional similarity of ABP 959 with eculizumab RP contributes to the TOE for biosimilarity and provides support for extrapolation based on inhibition of C5 function to other rare disease indications approved for eculizumab RP.

**Supplementary information:**

The online version contains ﻿supplementary material available at 10.1007/s00277-023-05439-4.

## Introduction

ABP 959 is being developed as a biosimilar to Soliris® (eculizumab) reference product (RP) [1, 2]. Eculizumab RP is a recombinant, humanized, monoclonal IgG subclass 2/4 kappa (IgG2/4к) antibody and binds to the human complement protein C5 with high affinity, thereby inhibiting its cleavage to C5a and C5b and preventing the generation of the terminal complement complex C5b-9 (also known as multiple attack complex; MAC) and blocking complement-mediated cell lysis.

Eculizumab RP is approved for four rare disease indications: paroxysmal nocturnal hemoglobinuria (PNH), atypical hemolytic uremic syndrome (aHUS), generalized myasthenia gravis (gMG), and neuromyelitis optica spectrum disorder (NMOSD), all under orphan designation [3, 4]. Though often difficult, the drug development process for rare disease therapeutics can result in revolutionary products which become the sole first-line therapy; such is the case for eculizumab RP and diseases related to the complement system. Eculizumab RP was first approved in 2007 by the FDA for the treatment of PNH, making it the first approved therapeutic specifically for the treatment of PNH. Prior to its approval, patients were managed based on handling symptoms of this rare and life-threatening hemolytic blood disorder using a variety of supportive care practices including a reliance on blood transfusions. PNH is particularly devastating because onset occurs in early adulthood, and the median survival is only 10–15 years post-diagnosis. Eculizumab RP significantly reduces complement activation and intravascular hemolysis, thus reducing PNH-related life-threatening morbidities [5].

Eculizumab RP was also the first approved treatment for the rare diseases, aHUS, NMOSD, and gMG [1, 2, 5], revolutionizing the care of these patients. aHUS is a condition that causes the formation of blood clots in the small blood vessels of the kidneys and is marked by the destruction of red blood cells, low platelet count, and kidney failure, with death rates as high as 25%. NMOSD is an autoimmune demyelinating disease of the central nervous system, characterized by inflammation of the optic nerve and spinal cord that results in demyelination that can lead to paralysis and blindness [6]. gMG is a chronic and progressive autoimmune neuromuscular disease. Inflammation at the neuromuscular synapse results in the disruption of the normal communication between nerves and muscles. gMG patients exhibit weakened muscles in the head, neck, limbs, and trunk including the respiratory muscles.

**Fig. 1:**
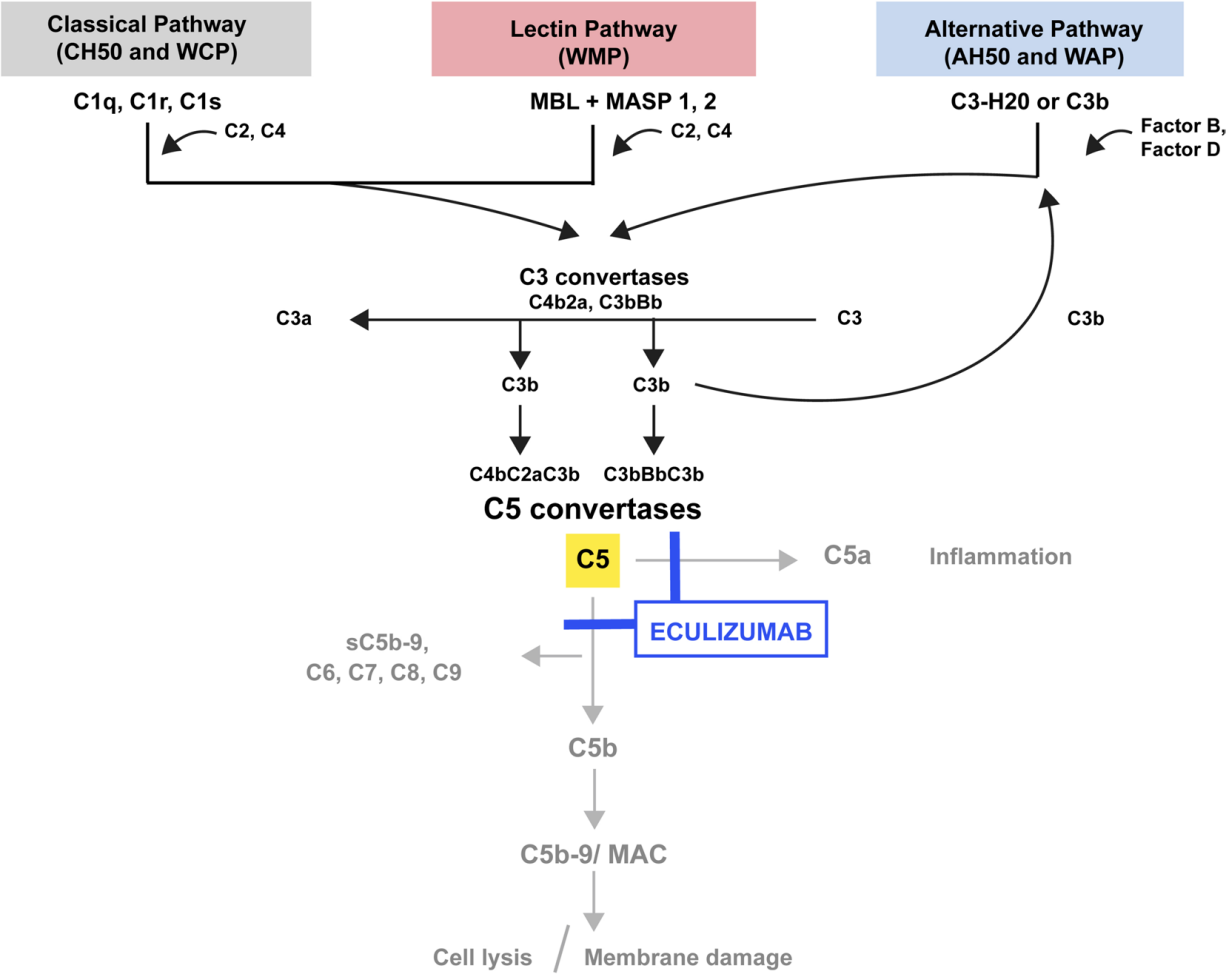
Complement pathway overview. Complement can be activated through three pathways: classical, lectin, and alternative. The classical pathway is activated when C1q, C1r, and C1s cleave C4 and C2. The lectin pathway is activated when mannose-binding lectin (MBL) and the MBL-associated serine proteases (MASPs) 1, 2 cleave C4 and C2. C4 and C2 cleavage products form the classical and lectin pathway C3 convertase, C4b2a, which cleaves C3 into C3b and C3a. The alternative pathway is activated when C3H2O or C3b and factors B and D, leading to the formation of the C3 convertase, C3bBb. All three pathways culminate in the formation of the C5 convertases, C4bC2aC3b (from the classical and lectin pathway) and C3bBbC3b (from the alternative pathway), which in turn generate the major effectors of the complement system, including the membrane attack complex (MAC), leading to cell lysis, membrane damage, and inflammation. Eculizumab inhibits the activation of the complement protein C5, blocking the formation of the MAC. CH50 and WCP assay the classical pathway, WMP assays the lectin pathway, and AH50 and WAP assay the alternative pathway

PNH, aHUS, NMOSD, and gMG are diseases of dysregulation of the complement system and are all improved by eculizumab inhibiting the generation of the MAC. The complement system is a major component of the innate immune system, and one of the first lines of defense against pathogenic microorganisms that breach the mechanical and chemical barriers of the body. Activation of the complement system occurs via three pathways: the classical pathway, alternative pathway, and lectin (or mannose binding) pathway [7]. All three pathways lead to a common effector pathway which culminates in the components C5b, C6, C7, C8, and C9 combining to form the MAC which causes cell lysis (Fig. 1). The classical pathway is initiated by the binding of the C1 complex (C1q, C1r and C1s) to bound antigen–antibody (IgG/IgM) complexes. The lectin pathway is activated through recognition of non-host glycosylation or sugar patterns. The alternative complement pathway occurs by the spontaneous hydrolysis of C3 or importantly in response to C3b formation caused by activation of either the classical or lectin pathways. It has been demonstrated that the alternative complement pathway may account for 80% of complement activation in vitro [8], because it serves as a hub regardless of where activation originates.

A biosimilar is a biological product that is highly similar to its RP, with no clinically meaningful differences. FDA-approved biosimilars increase the number of biologic treatment options and have the potential to decrease healthcare costs [9]. In the US, savings to the healthcare system have been estimated to be $54 billion over a 10-year period [10]. The scientific justification for use of ABP 959 as a biosimilar is being built on the totality of evidence (TOE) including analytical similarity [11] as well as pharmacokinetic (PK), pharmacodynamic (PD), and safety and immunogenicity similarities [12, 13], and a common mechanism of action (MoA) across indications, in this case the binding and inhibition of C5.

The concept of extrapolation is unique to biosimilar development and allows for the clinical use of the biosimilar in all indications for which the RP has been approved, without the requirement of comparative clinical trials in each indication. The support for scientific justification of a biosimilar product to extrapolated indications can be complex and includes data gathered from all relevant published assessments of the MoA, PK, PD, biodistribution, and expected toxicities, safety, and immunogenicity risk assessment across indications [14].

To generate additional substantive scientific justification for the extrapolation of ABP 959 to all approved indications of eculizumab RP beyond PNH, which is currently being evaluated in the ABP 959 confirmatory comparative clinical study in patients with PNH [12], we have generated human ex vivo simulated disease models of complement activation for aHUS and NMOSD. Thus, the work presented here uses multiple hemolysis and enzyme-linked immunosorbent assay (ELISA) based methods across complement activation pathways, to further support the functional similarity of ABP 959 and eculizumab RP and the extrapolation to all approved indications of eculizumab RP.

## Materials and methods

### Reagents

Pooled normal human sera were obtained from healthy individuals who had previously been tested for complement status under appropriate Institutional Review Board (IRB) approval (COMIRB #17–1183) at the University of Colorado. For the two hemolytic methods, CH50 and AH50, sheep and rabbit red blood cells and anti-sheep hemolysin were obtained from Colorado Serum Company. For the Wieslab complement function testing, individual kits were purchased from Svar Life Sciences (CP, COMPL CP310 RUO; AP, CAMPL AP330 RUO; and LP, COMPLMP320 RUO). The MicroVue sC5b-9 Plus EIA kits were purchased from Quidel Corporation. The ABP 959 reference standard, manufactured by Amgen, was used to optimize each method and to conduct dose-range finding studies in the two hemolytic methods in normal human serum before generating the simulated disease models. As this was only optimization work, the data are not presented here. Eculizumab (US) and eculizumab (EU) were sourced from US and EU markets, and ABP 959 was manufactured by Amgen. Health authorities require a comparison with the approved product within their region; thus two sources of eculizumab RP were used that represent the approved versions of the product in most of the world. This study aims to support similarity between eculizumab US and eculizumab EU, thus establishing a scientific bridge that would allow using a single RP comparator in future studies. Three lots each of eculizumab (US), eculizumab (EU), and ABP 959 were evaluated in each method in normal human serum, simulated aHUS serum, and simulated NMOSD serum.

### Simulated aHUS serum

Simulated aHUS serum was created to mirror the behavior of the complement pathway in patients with aHUS, since there is very limited access to aHUS patient samples. Autoantibodies to complement factor H accounts for around 10% of the cases of aHUS [15]. Therefore, to create simulated aHUS serum and get around the low availability of clinical samples, exogenous anti-factor H antibodies were added to normal serum. The simulated aHUS serum was prepared to mirror factor H inhibition in these patients using anti-factor H antibodies that have previously been shown to appropriately mimic the clinically relevant blocking of the function of factor H [16]. To prepare simulated aHUS serum, a 25 µg/mL amount of the anti-factor H antibody, OX 24 (Thermo Fisher Scientific) was added to normal human serum at a level that can be shown to inhibit factor H function by at least 30% [16]. Generally, the anti-factor H antibody, OX 24, inhibits Factor H function by approximately 50–60% (Online resource 1); the 30–60% inhibition represents a physiologically relevant range of inhibition observed in aHUS patients with Factor H deficiency [16].

### Simulated NMOSD serum

Simulated NMOSD serum was created to mirror the behavior of the complement pathway in patients with NMOSD, since there is very limited access to NMOSD patient samples. To prepare simulated NMOSD serum, a 300 µg/mL amount of antibody to AQP4 (anti-AQP4; Sigma) and a 3800 µg/mL amount of recombinant AQP4 protein (NBP2-52872PEP; NOVUS) were added to normal human serum to generate antibody complexes for a common auto-antigen. Patients with active NMOSD have increased concentrations of terminal complement components, such as soluble sC5b-9 in the cerebrospinal fluid [17]. The simulated NMOSD serum showed a dose-dependent increase of the terminal pathway complement activation, with sC5b-9 levels almost five times over the levels measured in the normal human serum after incubation at 37 °C for 30 min (Online resource 2).

### CH50 hemolytic method

This classical pathway functional assay was performed per the method of Kabet and Mayer [18]. In short, a functional hemolytic assay representing classical complement activation measures the concentration of serum necessary to lead to 50% complement hemolytic activity (CH50) based on lysis of a set concentration of antibody-coated sheep red blood cells (sEA). The readout of this assay is the amount of hemoglobin that is released when the coated red blood cells are lysed. Serial dilutions of normal human serum, simulated aHUS serum, or simulated NMOSD serum were prepared in Gelatin-Veronal Buffer containing calcium and magnesium. Equal volumes of prepared sEAs were then added; serum and cells were incubated together at 37 °C for 30 min. In parallel, control tubes for determination of 100% and 0% lysis were also prepared. At the end of the incubation period, 2 mL of normal saline was added, and the intact cells were removed by centrifugation. The amount of hemoglobin released into the supernatant from complement mediated cell lysis of the red blood cells was measured in a spectrophotometer at 415 nm [19].

### AH50 hemolytic method

The alternative pathway function assay, AH50, is similar to the CH50 assay but is based on the direct lysis of rabbit red blood cells (rEA) using chelation of calcium to keep the classical pathway inactive [20]. Specifically, activation of the calcium-dependent classical pathway was prevented by addition of 8 mM EGTA to chelate Ca^2+^ (the C1 complex is calcium dependent). The incubations and readout were the same as the classical pathway CH50.

### Wieslab classical pathway function enzyme-linked immunosorbent assay (ELISA)

The Wieslab classical pathway (WCP) product, COMPL CP310 RUO, was optimized and qualified by Exsera BioLabs. The manufacturer’s protocol indicates assay analysis can utilize a single point positive control (PC) and single negative control (NC) into a ratio of (Result-NC)/(PC-NC) × 100 or with a standard curve utilizing the PC to create the curve. The College of American Pathologists requires a minimum of a five-point curve and such recommendations are also included in FDA guidance documents [21]; thus, a six-point non-zero standard curve method was utilized to generate assay results. The WCP assay is based on a plate-coated immunoglobulin-based activator of the classical pathway. If the classical pathway is functional in the specimen, the complement cascade is activated and leads to the formation of a neo-epitope on the C5-C9 complex. The neo-epitope on the C5-C9 complex is then recognized by an included antibody in an ELISA format.

### Wieslab alternative pathway function ELISA

The Wieslab alternative pathway (WAP) product, COMPL CP330 RUO, was optimized and qualified by Exsera BioLabs. The manufacturer’s protocol indicated data analysis can utilize a single point PC and single NC into a ratio of (Result-NC)/(PC-NC) × 100; no mechanism for utilization of a full standard curve is given by the manufacturer. The College of American Pathologists requires a minimum of a five-point curve and such recommendations are also included in FDA guidance documents [21]; thus, a six-point non-zero standard curve was created, characterized, and utilized to generate assay results. The WAP assay is based on a plate coated with a lipopolysaccharide-based activator of the alternative pathway. If the alternative pathway is functional in the specimen, the complement cascade is activated, leading to the formation of a neo-epitope present on the C5-C9 complex. The neo-epitope on the C5-C9 complex is then recognized by an included antibody in an ELISA format. When using the manufacturer's ratio method, the lowest level reported was zero.

### Wieslab mannose binding lectin (MBL) pathway function ELISA

The Wieslab mannose activation assay (WMP) product, COMPL CP320 RUO, was optimized by Exsera BioLabs. The manufacturer protocol indicated data analysis can utilize a single point PC and single NC into a ratio of (Result-NC)/(PC-NC) × 100; no mechanism for utilization of a full standard curve is given by the manufacturer. The College of American Pathologists requires a minimum of a five-point curve and such recommendations are also included in FDA guidance documents [21]; thus, a six-point non-zero standard curve was created, characterized, and utilized to provide assay results. The WMP assay is based on a plate coated with a mannose-based activator of the lectin/MBL pathway. If the lectin pathway is functional in the specimen, the complement cascade is activated, leading to the formation of a neo-epitope present on the C5-C9 complex recognized by an included antibody that binds to a neo-epitope C9 present only when in this complex. To ensure the assay is specific for the lectin pathway, the classical pathway is blocked with a neutralizing antibody against C1q [22–24].

### sC5b-9 soluble membrane attack complex by ELISA

The sC5b-9 ELISA was performed according to the manufacturer’s protocol (Quidel sC5b-9 Plus EIA). The sC5b-9 assay utilizes a plate-coated mouse monoclonal antibody that binds to C9 to capture sC5b-9 complexes. The captured sC5b-9 complexes are measured by a chromogenic enzyme substate that reacts with horseradish peroxidase-conjugated antibodies bound to antigens present in the complex. The amount of absorbance was measured in a spectrophotometer at 450 nm. The color intensity of the reaction mixture was proportional to the concentration of sC5b-9 present in the test specimens, standards, and controls.

### In vitro test article incubation

For all methods examining normal human and simulated aHUS serum, ABP 959, eculizumab US, and eculizumab EU test articles were prepared at 10 different concentrations ranging from 10 to 200 µg/mL and then combined with a set pool of serum (either normal or simulated aHUS) at a ratio of 1 to 9. The serum and test article were then incubated 10 min at 37 °C prior to proceeding with multiple assessments of complement activity as described above.

For all methods examining simulated NMOSD serum, ABP 959, eculizumab US, and eculizumab EU test articles were prepared at 8 different concentrations ranging from 9 to 150 µg/mL and then combined with a set pool of simulated NMOSD serum at a ratio of 1 to 9. The simulated NMOSD serum and test article were then incubated 30 min at 37 °C prior to proceeding to the above-described complement-activity assessments.

## Results

1. ABP 959 and eculizumab RP exhibit similar inhibition of the classical and the alternative complement activation pathways in normal human, simulated aHUS, and simulated NMOSD sera

To compare ABP 959 and eculizumab RP inhibition of complement activity in normal human serum as well as in disease-simulated samples, standard hemolytic methods for the classical (CH50) and alternative pathways (AH50) were evaluated. An in vitro dose-range finding study was first conducted using ABP 959 Reference Standard (RS) to optimize these hemolytic methods using normal human serum.

In the CH50 assay, a concentration range from 10 to 200 µg/mL ABP 959 RS in normal human serum and simulated aHUS serum was established upon determining maximal inhibition of hemolysis at around 40 µg/mL ABP 959 RS (Online Resource 3 and Online Resource 4). The optimal test concentration range for simulated NMOSD serum was determined to be 9 to 150 µg/mL (Online Resource 5).

**Fig. 2:**
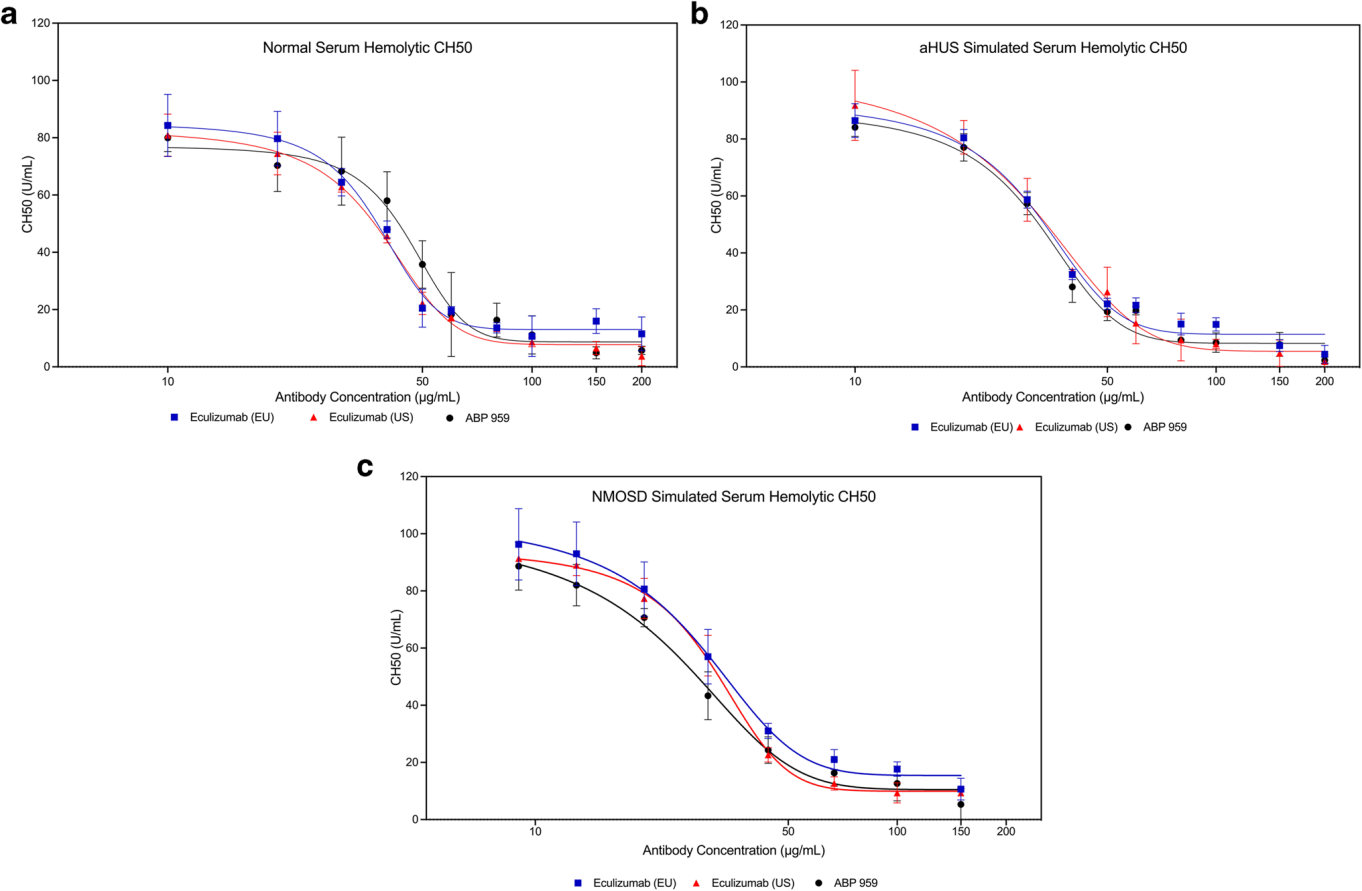
Inhibition of the classical complement pathway using the CH50 hemolytic method using three lots each of ABP 959, eculizumab (EU) and eculizumab (US) in normal human serum (**a**), simulated aHUS serum (**b**), and simulated NMOSD serum (**c**)

Once concentration ranges were determined, the inhibition of the classical complement pathway using the CH50 method was tested for all three anti-C5 antibodies in a side-by-side manner. Three lots each of eculizumab (US), eculizumab (EU), and ABP 959 were compared in normal serum, simulated aHUS serum, and simulated NMOSD serum. Similar, dose dependent inhibition of the classical pathway was observed in all three serum types. Figure 2a, b, and c represents the data from the mean of three lots for each test article and show similar activity of eculizumab (US), eculizumab (EU), and ABP 959.

Using ABP 959 RS for the dose-finding approach in the AH50 method, maximal inhibition of the alternative complement pathway in normal human serum was also observed using 40 µg/mL ABP 959 RS. Thus, the eculizumab RP dose range for this assay was also set at 10–200 ug/mL. Unlike the CH50 assay, a narrower range of hemolysis across the dose-curve was observed in all serum types using the AH50 method (Online Resources 3–5) [25].

**Fig. 3:**
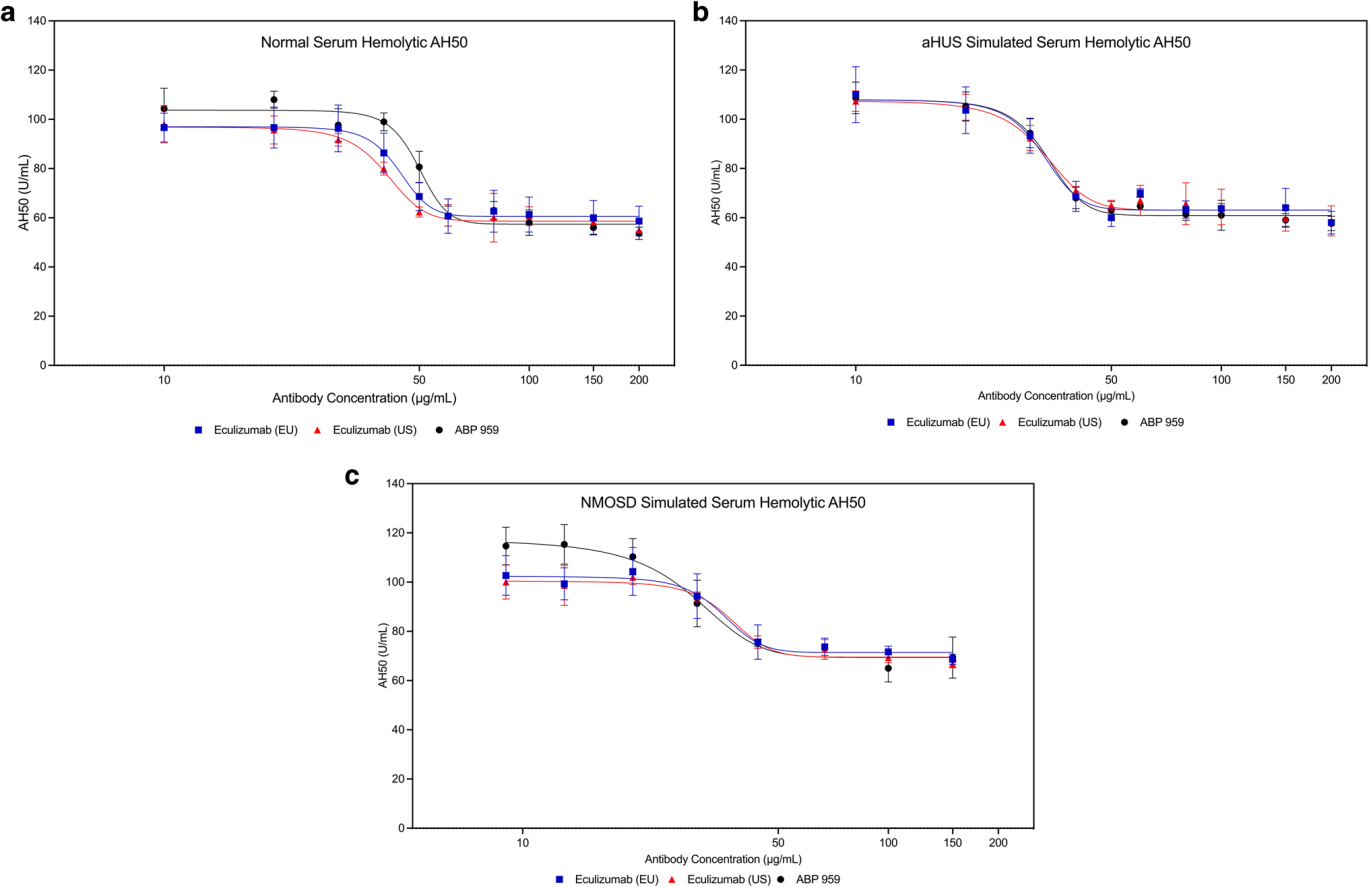
Inhibition of the alternative complement pathway using the AH50 hemolytic method using three lots each of ABP 959, eculizumab (EU) and eculizumab (US) in normal human serum (**a**) simulated aHUS serum (**b**) and simulated NMOSD (**c**)

Once concentration ranges were determined, the inhibition of the alternative complement pathway using the AH50 method was tested for all three anti-C5 antibodies in a side-by-side manner. Three lots each of eculizumab (US), eculizumab (EU), and ABP 959 were compared in normal serum, simulated aHUS serum, and simulated NMOSD serum. Similar, dose-dependent inhibition of the alternative pathway was observed in all three serum types (Fig. 3a, b, and c).

2. ABP 959 and eculizumab RP exhibit similar inhibition of the lectin pathway using normal human, simulated aHUS, and simulated NMOSD sera

**Fig. 4:**
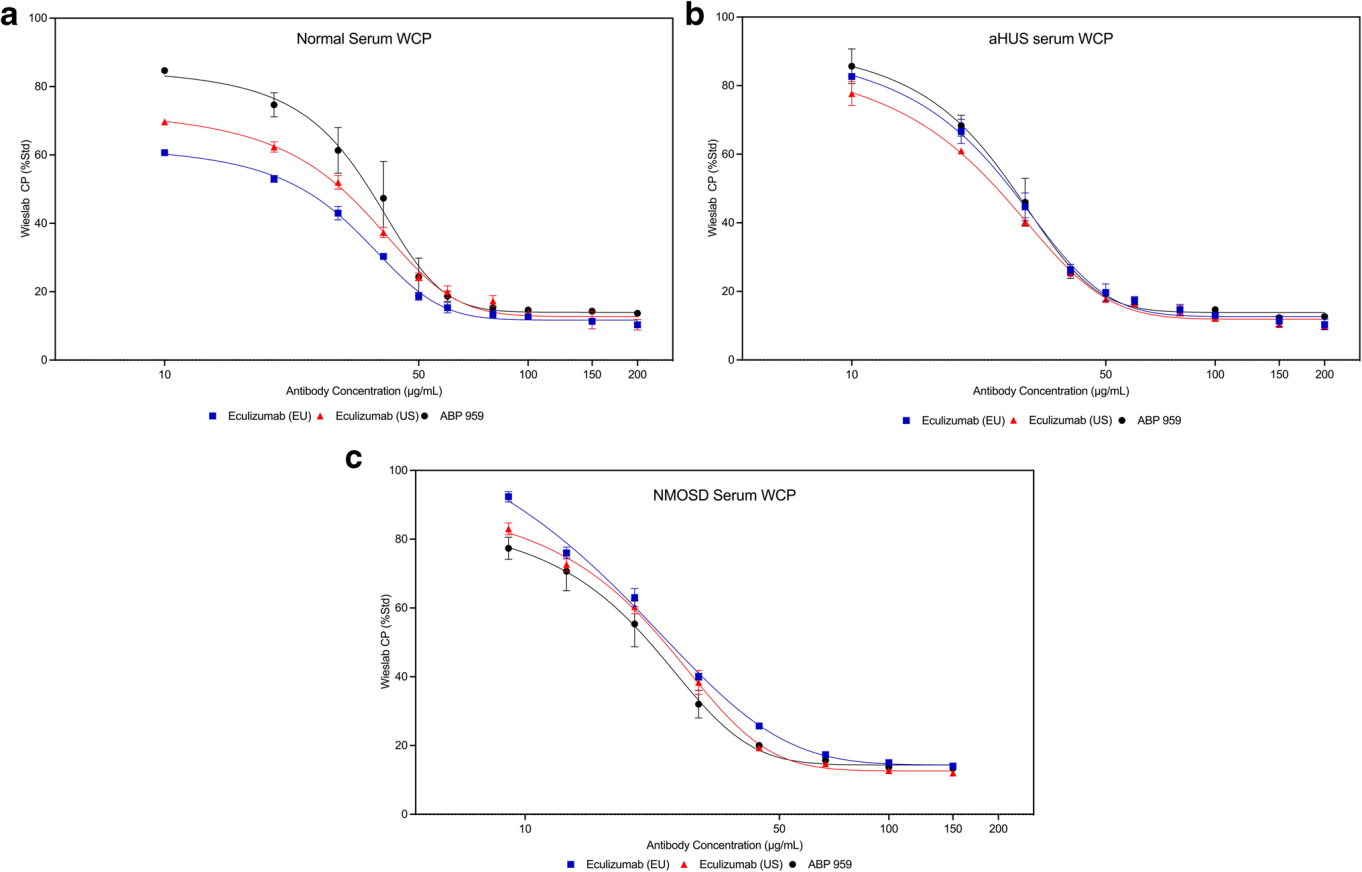
Inhibition﻿ of the classical complement pathway by the Wieslab method (WCP) using three lots each of ABP 959, eculizumab (EU) and eculizumab (US) in normal human serum (**a**), simulated aHUS serum (**b**), and simulated NMOSD serum (**c**)

**Fig. 5:**
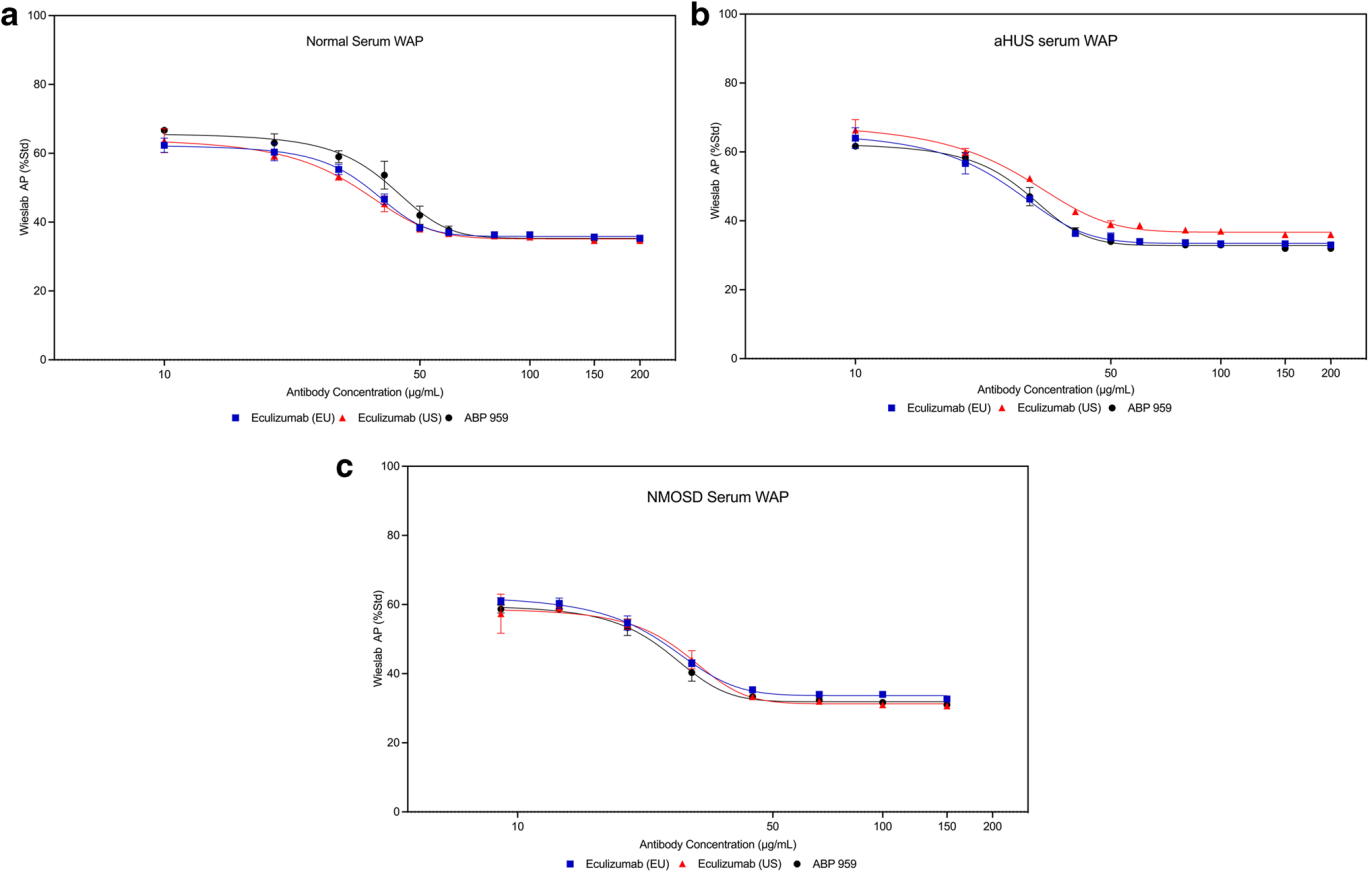
Inhibition of the alternative complement pathway by the Wieslab method (WAP) using three lots each of ABP 959, eculizumab (EU) and eculizumab (US) in normal human serum (**a**), simulated aHUS serum (**b**), and simulated NMOSD serum (**c**)

**Fig. 6:**
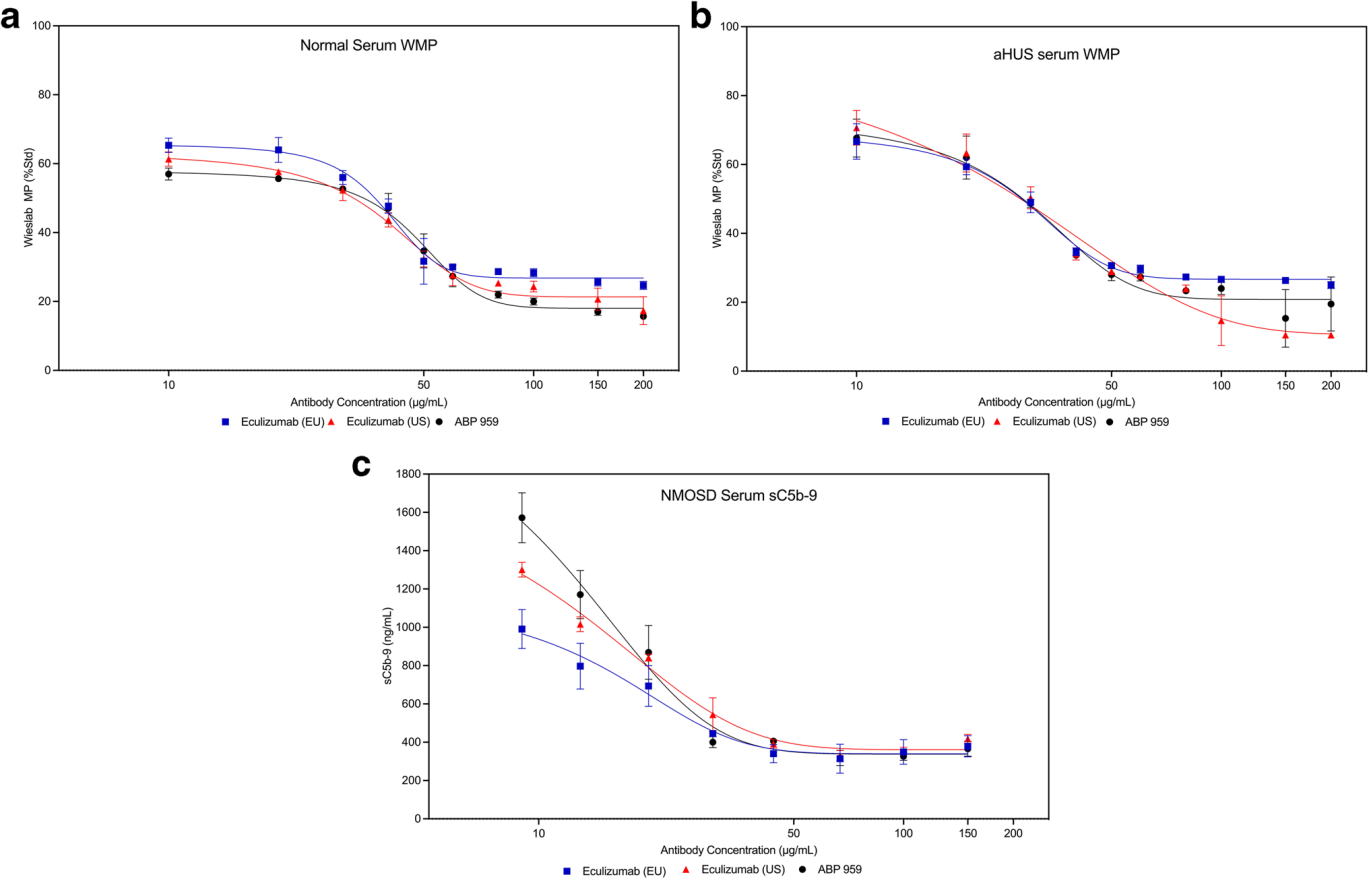
Inhibition ﻿of the mannose-binding lectin complement pathway by the Wieslab method (WMP) using three lots each of ABP 959, eculizumab (EU) and eculizumab (US) in normal human serum (**a**) and simulated aHUS serum (**b**). For simulated NMOSD serum (**c**) inhibition was observed by sC5b-9 assay using three lots each of ABP 959, eculizumab (EU) and eculizumab (US)

While CH50 and AH50 are hemolytic methods often used in testing complement abnormalities [26], there is no available method to address the lectin pathway using a hemolysis endpoint. To demonstrate whether ABP 959 and eculizumab RP exhibit similarities in inhibiting the lectin pathway, we utilized the Wieslab ELISA-based methods panel to assess the lectin pathway in addition to the classical and alternative pathways. Multiple doses of three lots each of eculizumab (US), eculizumab (EU), and ABP 959 were compared in the Wieslab ELISA-based methods panel using normal serum, simulated aHUS serum, and simulated NMOSD serum. Dose-dependent inhibition of the classical pathway (WCP) was observed in normal human serum (Online Resource 3 and Fig. 4a), aHUS serum (Online Resource 4 and Fig. 4b), and NMOSD serum (Online Resource 5 and Fig. 4c). Dose-dependent inhibition of the alternative pathway (WAP) was observed in normal human serum (Online Resource 3 and Fig. 5a), aHUS serum (Online Resource 4 and Fig. 5b), and NMOSD serum (Online Resource 5 and Fig. 5c). Dose-dependent inhibition of the lectin pathway (WMP) was observed in normal human serum (Online Resource 3 and Fig. 6a) and aHUS serum (Online Resource 4 and Fig. 6b). NMOSD serum was additionally tested in the membrane-attack-complex-formation (sC5b-9) assay where dose-dependent inhibition was observed as well (Online Resource 5 and Fig. 6c).

In summary, similar, dose-dependent inhibition of the classical, alternative, and lectin pathways was observed in all three serum types using ELISA.

## Discussion

ABP 959 is being developed as a biosimilar for eculizumab RP and was evaluated in a confirmatory comparative clinical study only in patients PNH [12]. To generate additional scientific justification for extrapolation to all approved indications of eculizumab RP beyond PNH, we utilized ex vivo human-based complement assays to evaluate inhibition of the complement pathway in the additional approved indications of aHUS and NMOSD. To this end, ex vivo comparison of ABP 959 and eculizumab RP in multiple functional hemolysis and Wieslab ELISA methods for complement inhibition was performed using normal human serum, simulated aHUS serum, and simulated NMOSD serum. The results provided here demonstrate similar inhibitory activity of ABP 959 and eculizumab RP in all assays and conditions tested, supporting the scientific justification for extrapolation to all approved indications of the eculizumab RP, including but not limited to aHUS and NMOSD.

Since both aHUS and NMOSD are rare genetic disorders with limited patient populations, serum sample collection from patients with these disorders is challenging and can present ethical concerns given the nature of the disorders. Patients suffering from rare genetic disorders can be hard to identify especially early on in their disease progression, geographically dispersed, and are often children [27]. When exposure–response data for the RP is unknown, small clinical studies can be conducted to generate the necessary information, but in the case of rare diseases such as aHUS and NMOSD treated with eculizumab RP, modeling and simulation become particularly useful essential tools in characterizing the PK profiles, PD, and the dose-exposure relationship. Therefore, we developed simulated serum models based on the mechanism of complement dysfunction causing each disease. Simulated aHUS serum was generated by adding neutralizing antibodies against factor H to mimic the depletion of this inhibitory factor in patients with aHUS. Simulated NMOSD serum was created by adding preformed antibody complexes against AQP4, a common auto-antigen in NMOSD patients [28]. A combination of hemolytic (CH50 and AH50) and Wieslab ELISA methods were used to evaluate all three complement pathways using normal sera, simulated aHUS sera, and simulated NMOSD sera. CH50 and AH50 hemolysis methods assess the functional activities between ABP 959 and eculizumab RP to inhibit classical and alternative complement pathways, respectively. ABP 959 and eculizumab RP inhibited > 90% of hemolysis in both the CH50 and AH50 hemolysis assays over a concentration range of 50 to 100 µg/mL supporting the conclusion that ABP 959 functions in a way similar to eculizumab RP in inhibiting both the classical and alternative complement pathways. ELISA-based methods also demonstrated that ABP 959 and eculizumab RP dose-dependently inhibit complement formation in normal, simulated aHUS, and simulated NMOSD serum to a similar degree. These data complement and further support the use of such simulated serum models in rare diseases and assays to model clinical performance of anti-C5 antibodies in an ex vivo setting, an important advantage given the challenges of developing therapeutics for rare complement diseases.

Interestingly, there are a limited number of reports directly comparing the activity of anti-C5 antibodies, such as eculizumab, across test methods using hemolysis and ELISA-based readouts. Our results demonstrate that these methods are comparable in our hands using the same anti-C5 antibody dose range, even to reproducing the plateau in inhibition for the alternative pathway that has been observed using hemolysis as an endpoint. Further, the performance of both assay types (hemolysis and ELISA) is robust across simulated conditions for aHUS and NMOSD, suggesting the use of ELISA-based methods as a viable alternative for hemolysis assays.

In summary, the results presented in this study add to the totality of scientific evidence for the extrapolation of ABP 959 to all approved indications for eculizumab RP, including but not limited to aHUS and NMOSD. We have previously established analytical similarity [11] as well as PK similarity between ABP 959 and eculizumab RP [13]. In other approved indications for eculizumab RP, a similar PK is also expected between ABP 959 and eculizumab RP based on the current weight-based dosing approach in patients [29]. In addition, comparative clinical studies demonstrate PK, PD, and safety and immunogenicity similarity of ABP 959 with eculizumab RP [12, 13]. Finally, clinical data in both healthy adults [13] and adult patients with PNH [12] support the conclusion that there are no clinically meaningful differences between ABP 959 and eculizumab RP. Thus, results from this work provide new additional evidence that the ability of ABP 959 and eculizumab RP to inhibit C5 in normal human serum, simulated aHUS serum, and simulated NMOSD serum is similar. The addition of biosimilar therapies has the potential to offer doctors more treatment options, patients access to lower cost therapeutics, facilitate more equitable health outcomes, and as a result, reduce overall health care costs [30].

## Conclusions

In summary, our data support that ABP 959 is highly similar to eculizumab RP using multiple ex vivo assays evaluating the classical, alternative, and lectin complement pathways across 3 conditions: normal human serum, simulated aHUS serum, and simulated NMOSD serum [31]. This work, along with the totality of evidence supporting similarity of ABP 959 with eculizumab RP, provides the scientific rationale and support for extrapolation for all indications approved for eculizumab RP, including but not limited to aHUS and NMOSD.

### Supplementary information

Below is the link to the electronic supplementary material.

Supplementary file1 (PDF 192 KB)

## Data Availability

NA.
