# Supplementary material for: Functional similarity of ABP 959 and eculizumab in simulated serum models of aHUS and NMOSD
Source: Ann Hematol. 2023 Oct 10;102(12):3299–309. doi: 10.1007/s00277-023-05439-4 (PMC10640484; doi:10.1007/s00277-023-05439-4)
Supplement: Supplementary file 1 — Supplementary file1 (PDF 192 KB) [file 277_2023_5439_MOESM1_ESM.pdf]

ONLINE RESOURCE

Functional similarity of ABP 959 and eculizumab in simulated serum model of aHUS and NMOSD

Helen J. McBride[1], Ashley Frazer-Abel[2], Sandra Thiemann[1], Sonya G. Lehto[1], Katariina M. Hutterer[1], and Jennifer Liu[1]

[1]Amgen Inc., One Amgen Center Dr., Thousand Oaks, CA 91320

[2]University of Colorado, School of Medicine, Aurora, CO 80045

Online Resource 1. Level of Inhibition of Factor H Function by the Addition of anti-Factor H Antibody, OX 24.

	Factor H Function (U/mL)	% Reduction with OX 24
Normal serum	24	59%
Serum + anti-factor H antibody (OX 24)	58	

Online Resource 2. Dose Dependent Increase of Complement Activation in Simulated NMOSD Serum.

Abbreviation: NMOSD, neuromyelitis optica spectrum disorder.

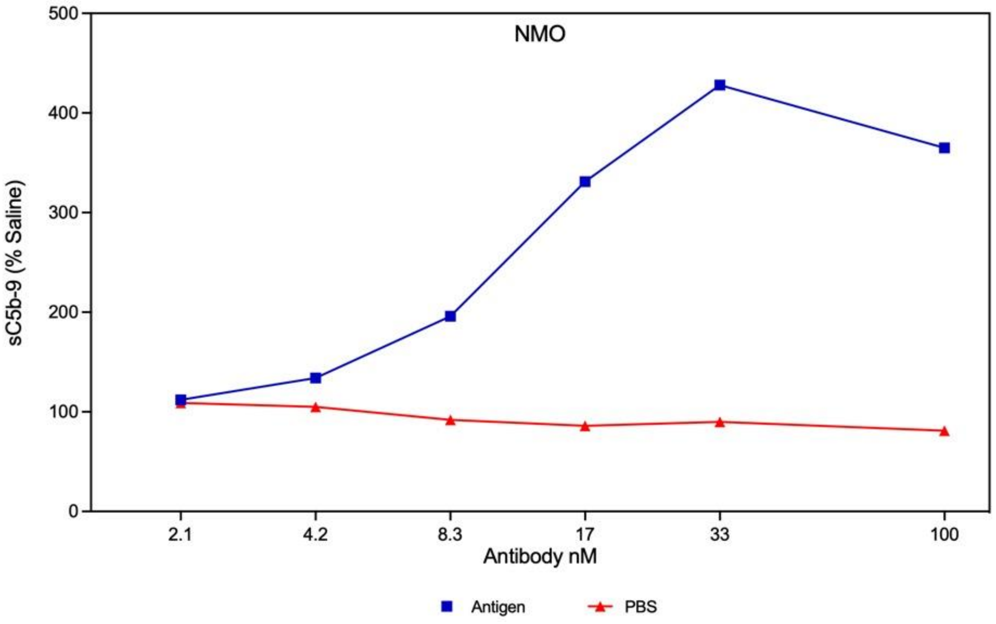

Online Resource 3. Complement Pathway Inhibition by ABP 959, Eculizumab (EU) and Eculizumab (US) in Five Complement Pathway Assays Using Normal Human Serum.

	Antibody Concentration (µg/mL)	Eculizumab (EU) Mean (U/mL)	Eculizumab (US) Mean (U/mL)	ABP 959 Mean (U/mL)
CH50	10	84.31	80.89	79.97
	20	79.67	74.46	70.33
	30	64.47	62.86	68.35
	40	47.95	45.74	58.00
	50	20.49	22.19	35.77
	60	19.66	17.29	18.26
	80	13.63	13.39	16.33
	100	10.68	8.65	11.16
	150	15.95	6.55	4.90
	200	11.53	3.72	5.74
AH50	10	96.67	98.00	104.33
	20	96.67	95.67	108.00
	30	96.33	91.67	97.67
	40	86.33	80.00	99.00
	50	68.67	62.33	80.67
	60	60.67	61.00	60.67
	80	62.67	60.00	63.00
	100	61.33	60.67	58.00
	150	60.00	58.33	56.00
	200	58.67	55.00	53.67
WCP	10	60.67	69.67	84.67
	20	53.00	62.33	74.67
	30	43.00	52.00	61.33
	40	30.33	37.33	47.33
	50	18.67	24.33	24.33
	60	15.33	20.00	18.67
	80	13.33	17.33	15.33
	100	12.67	13.67	14.67
	150	11.33	11.67	14.33
	200	10.33	10.33	13.67
WAP	10	62.33	63.67	66.67
	20	60.33	59.33	63.00
	30	55.33	53.33	59.00
	40	46.67	45.33	53.67
	50	38.33	38.00	42.00
	60	37.00	36.67	37.67
	80	36.33	36.00	36.33

	100	36.33	35.67	36.00
	150	35.67	34.67	35.00
	200	35.33	34.67	34.67
WMP	10	65.33	61.33	57.00
	20	64.00	57.67	55.67
	30	56.00	52.33	52.67
	40	47.67	43.67	47.00
	50	31.67	32.33	34.67
	60	30.00	27.67	27.33
	80	28.67	25.33	22.00
	100	28.33	24.33	20.00
	150	25.67	20.67	17.00
	200	24.67	17.33	15.67

Abbreviations: AH50, alternative pathway hemolytic 50; CH50, classical pathway hemolytic 50; eculizumab (EU), eculizumab reference product sourced from the European Union; eculizumab (US), eculizumab reference product sourced from the United States; WAP, Wieslab alternative pathway; WCP, Wieslab classic pathway; WMP, Wieslab MBL/Lectin pathway.

Online Resource 4. Complement Pathway Inhibition by ABP 959, Eculizumab (EU) and Eculizumab (US) in Five Complement Pathway Assays Using Simulated aHUS Serum.

	Antibody Concentration (μg/mL)	Eculizumab (EU) Mean (U/mL)	Eculizumab (US) Mean (U/mL)	ABP 959 Mean (U/mL)
CH50	10	86.43	91.78	84.10
	20	80.40	80.60	77.05
	30	58.69	58.66	57.33
	40	32.43	33.82	28.09
	50	22.11	26.34	19.37
	60	21.63	15.35	19.79
	80	15.04	9.44	9.41
	100	14.95	8.07	8.54
	150	7.51	4.80	7.91
	200	4.46	1.89	2.33
AH50	10	110.00	107.33	108.67
	20	103.67	104.67	105.33
	30	93.33	92.33	94.33
	40	68.67	71.00	68.00
	50	60.00	65.00	63.00
	60	70.00	67.00	64.67
	80	63.33	65.67	61.33
	100	63.67	64.33	61.00
	150	64.00	59.67	59.00
	200	58.00	58.67	57.67
WCP	10	82.67	77.67	85.67
	20	66.67	61.00	68.33
	30	44.67	40.33	46.00
	40	26.33	25.33	25.33
	50	19.67	17.67	19.33
	60	17.33	16.33	17.00
	80	14.67	14.33	14.67
	100	13.00	12.33	14.67
	150	11.33	10.33	12.33
	200	10.33	10.00	12.67
WAP	10	64.72	66.28	61.48
	20	56.90	59.64	58.17
	30	46.29	52.30	47.00
	40	36.57	42.45	36.73
	50	35.42	39.04	33.81
	60	34.06	38.70	34.07
	80	33.36	37.08	32.86

	100	33.36	36.89	32.92
	150	33.34	36.13	32.29
	200	32.94	35.72	32.13
WMP	10	66.79	70.37	67.91
	20	59.38	63.68	62.02
	30	48.96	50.52	48.55
	40	34.38	34.21	33.48
	50	30.77	28.83	27.77
	60	29.69	27.40	27.63
	80	27.41	23.99	23.30
	100	26.45	14.65	24.10
	150	26.38	10.50	15.20
	200	25.19	10.50	19.39

Abbreviations: AH50, alternative pathway hemolytic 50; aHUS, atypical hemolytic uremic syndrome; CH50, classical pathway hemolytic 50; eculizumab (EU), eculizumab reference product sourced from the European Union; eculizumab (US), eculizumab reference product sourced from the United States; WAP, Wieslab alternative pathway; WCP, Wieslab classic pathway; WMP, Wieslab MBL/Lectin pathway.

Online Resource 5. Complement Pathway Inhibition by ABP 959, Eculizumab (EU) and Eculizumab (US) in Five Complement Pathway Assays Using Simulated NMOSD Serum.

	Antibody Concentration (μg/mL)	Eculizumab (EU) Mean (U/mL)	Eculizumab (US) Mean (U/mL)	ABP 959 Mean (U/mL)
CH50	9	96.36	91.30	88.54
	13	92.77	88.70	82.17
	20	80.66	77.35	70.31
	30	57.22	57.40	43.40
	44	30.85	22.54	24.53
	67	21.13	12.91	16.32
	100	17.57	9.39	12.62
	150	10.79	9.17	5.31
AH50	9	102.67	100.00	114.67
	13	99.33	98.67	115.33
	20	104.33	102.00	110.33
	30	94.33	94.00	91.33
	44	75.67	75.67	75.33
	67	73.67	72.67	73.33
	100	71.67	69.33	65.00
	150	68.67	66.67	69.33
WCP	9	92.45	82.84	77.29
	13	76.08	72.92	70.66
	20	63.02	60.27	55.50
	30	40.08	38.43	31.90
	44	25.64	19.49	19.97
	67	17.30	14.59	15.84
	100	15.07	12.57	13.72
	150	13.84	11.90	13.23
WAP	9	61.07	57.22	58.87
	13	60.25	58.55	58.85
	20	54.80	54.52	53.32
	30	43.39	44.00	39.99
	44	35.27	33.10	33.38
	67	34.08	32.04	32.44
	100	33.78	30.89	31.67
	150	32.56	30.66	31.25
sC5b-9	9	991.06	1300.86	1571.79
	13	796.82	1015.81	1170.79
	20	693.58	841.44	869.16
	30	445.09	544.27	400.15
	44	340.10	391.13	405.38

	67	313.85	328.96	318.20
	100	348.85	354.74	326.92
	150	*378.60*	*418.42*	*365.28*

Abbreviations: AH50, alternative pathway hemolytic 50; CH50, classical pathway hemolytic 50; eculizumab (EU), eculizumab reference product sourced from the European Union; eculizumab (US), eculizumab reference product sourced from the United States; NMOSD, neuromyelitis optica spectrum disorder; sC5b-9, soluble membrane attack complex; WAP, Wieslab alternative pathway; WCP, Wieslab classic pathway.
